# Progress in Medicine

**Published:** 1899-04-29

**Authors:** 


### Progress in Medicine.

### DISEASES OF THE RESPIRATORY SYSTEM.

*(Continued from page 417, Vol. XXV.)*

**Phthisis.**—Loomis[1] states that it is possible in some cases to diagnose phthisis previous to the appearance of physical signs, or of tubercle bacilli in the sputum. Weight, respiratory capacity, and chest measurement considered in relation to the height, furnish important aid. Dividing the weight expressed in pounds by the height in feet, a factor is obtained which in normal health is in man 26, in women 23. The average of two chest measurements, one taken during forced inspiration and the other during forced expiration, should never be less than half the height. In health there should be three cubic inches of vital capacity to every inch of height. Chloroanæmia and persistent and unexplained disturbances of the digestive system are symptoms of the pretuberculous stage of phthisis. The pulse in the pretuberculous stage is peculiar in that change of position has practically no influence in its rhythm, and in the relative feebleness of arterial pressure. Wolf Immerman[2] regards the variation in weight as one of the most important factors in the prognosis of phthisis. It is important to always weigh the patient at the same time of the day and to note how much food is being consumed. Without discoverable alteration in the physical signs or general condition there may be marked alteration in body weight, and these are to be attributed to psychical causes. Annoyance or excitement cause a fall in body weight, whilst a new departure in treatment or any added element of hope almost always cause a temporary rise.

Abnormal cardiac physical signs are sometimes met with in cases of phthisis. Thus in a case described by Willmore[3] in which there was advanced disease of the whole of the right lung and some slight affection of the left, no cardial impulse could be felt on the left side, and dulness was entirely absent. At the usual situation for the apex the heart sounds were almost inaudible, but were of normal intensity in the fifth right intercostal space 2¾ inches from the midsternal line; there also could be felt distinct impulse. The area of absolute cardiac dulness reached the fourth right intercostal space above and to within one inch of the right vertical nipple line. There was no evidence of fibrosis. Post-mortem examination showed the heart to be in no way displaced or enlarged. The left lung completely covered the heart to the midsternal line, whilst in the upper part of the right lung there were cavities, the remainder being solid and airless. The heart sounds had been conducted through this consolidated area, but the presence of the right-sided impulse is not so easily explained. A case closely resembling the above was recorded by Hale White in the *Lancet* of November 19th, 1898, and this observer arrived at the same conclusions with regard to the causation of the dextrocardia. Ewart,[4] however, maintains that in such cases the heart is really altered in position, but that the method of conducting the post-mortem examination hides this. In extensive disease of the right lung if the chest wall does not fall in then the space must be filled either by emphysematous distension of the right middle and lower lobes, or by the encroachment of the heart and left lung. Two such cases, one of phthisis and the other of bronchiectasis, have come under his notice. The displacement may be recognised at the post-mortem examination if the pericardium is opened from the abdomen before opening the chest, for then the pleuro-pericardial adhesions which are continuous with the retracted fibrous tissue of the lung are untouched. These instances in which the mobile heart is held in the right chest by atmospheric pressure owing to destructive changes in the right lung might be diagnosed with the help of skiagraphy. King[5] gives the following as the most reliable data upon which to base prognosis in consumption: Pulse and temperature, degree and rapidity of emaciation, condition of the organs of elimination, and condition of the digestive system. With regard to the pulse a small compressible pulse persisting above 120 is unfavourable, as is also a temperature reaching 102 deg. or 104 deg., accompanied with profuse diaphoresis. Increase in weight is generally a favourable sign, but occasionally exceptions are found to this rule. Acute nephritis is a serious complication, and chronic Bright's generally hastens a fatal termination. Dyspepsia materially contributes to a fatal ending. The quantity of expectoration and the number of tubercle bacilli therein have no prognostic significance whatever.

In view of the attention now being paid in this country to the open-air treatment of consumption and the establishment of sanatoria, the report of the old-established Loomis sanatorium[6] for the year ending November 1st, 1898, will be read with much interest. Of the 204 patients treated 28 are described as cured, there being no longer any apparent lesion of the lung or tubercle bacilli in the sputum. Six per cent. of patients discharged at the end of three months' stay were cured, and 11 per cent. of those discharged after a longer course of treatment. Sixty-seven of the 204 were "discharged improved," but the meaning of this term is not made clear. Progress was more marked during the winter than the summer months, no deleterious effects being noticed from exercise and constant living in the fresh air. Treatment with antituberculous serum manufactured by the United States Government has on the whole been encouraging, and has led to reduction of temperature, decrease in the number of bacilli, and possible immunity conferred upon patients after returning to their homes. Forty cases of tubercular laryngitis were treated, and 43 per cent. discharged cured.

[1] Med. Rec., Dec. 10, 1898. [2] Münc. Med. Woch, June 21, 1898. [3] Lancet, Dec. 10, 1898. [4] Lancet, Feb. 11, 1898. [5] Med. News, Dec. 3, 1898. [6] Med. News, Jan. 7, 1899.

